# Linking satellite-derived vegetation health parameters to arborist ground observations in urban environment

**DOI:** 10.1038/s41598-026-47953-1

**Published:** 2026-04-16

**Authors:** Lenka Foltýnová, Marek Fajstavr, Marko Stojanović, Martin Vokřál, Daniel Bicák, Sergei Mikhailov, Ondřej Nezval, Petr Horáček, Jan Labohý

**Affiliations:** 1 Global Change Research Institute, Czech Academy of Sciences, Bělidla 4a, Brno, 603 00 Czech Republic; 2 Faculty of Forestry and Wood Technology, Department of Wood Science and Wood Technology, Mendel University in Brno, Brno, Czech Republic; 3 ASITIS, s.r.o., Vážného 10, Brno, Czech Republic; 4 World from Space, Pellicova 3, Brno, Czech Republic; 5 Faculty of Science, Department of Applied Geoinformatics and Cartography, Charles University, Prague, Czech Republic

**Keywords:** Climate sciences, Ecology, Ecology, Environmental sciences

## Abstract

Trees in cities play a crucial role in regulating the urban environment and promoting citizens’ health and well-being. However, trees are under constant stress, causing reduction of provided ecosystem services, faster decline, and a need for intensive maintenance. Here, we test parameters derived from Enhanced Vegetation Index (EVI) that enables effective health and vitality assessment through satellite observation in two pilot cities (Lisbon and Copenhagen), and link them to arboricultural terms using data from ground surveys. Results show differences in the interpretation of EVI in contrasting environments. In Copenhagen, trees respond to stress through changes in chlorophyll content expressed by EVI, whereas in Lisbon, EVI primarily reflects variations in vitality. The newly introduced composite stress index shows strong agreement with arborist observations. These results highlight the EVI as a promising and cost-effective tool for assessing the health status of urban greenery, reducing the need for frequent and expensive ground observations.

## Introduction

Urban greenery is an essential element of sustainable and livable cities, providing ecosystem services such as temperature regulation, air filtering, carbon storage, stormwater regulation, and noise reduction^1–6^. In addition to these ecological functions, a wealth of research has linked exposure to trees and green spaces with significant benefits for citizens’ mental and physical well-being^7–10^. Urban trees and parks play a crucial role in public health, helping to reduce anxiety and depression^11,12^, promote active lifestyles^13^ and strengthen community health overall^14^.

However, establishing and maintaining trees in the city is challenging as urban environments impose harsh, often extreme, stress conditions^11^. Dense construction and paved surfaces restrict root growth and limit water availability, while pollution, traffic, and the urban heat island amplify environmental stresses^15^. Acting together, such stressors place chronic stress on urban trees and shortened tree lifespans compared to those in rural areas^16^. Street trees in particular suffer the greatest strain^17^ and may only live a fraction of their natural lifespan due to these compounded stresses^16^. With climate change driving more frequent heatwaves, droughts, and extreme weather events, these challenges are expected to intensify^18^. This not only threatens the health of the trees but also diminishes the valuable ecosystem services (e.g. shade, cooling, carbon sequestration or air purification) that healthy urban forests provide to city residents^19,20^.

Urban planners, landscape architects, and greenery maintenance workers must deal with a need for densification of greenery on one hand, and unfavorable conditions on the other hand, while often lacking exact information about the intensity of stress and the actual condition of the trees that are already in place. Compounding the challenge, the financial demands of maintaining and monitoring urban trees are substantial and growing^21,22^. Caring for thousands of street and park trees requires regular pruning, irrigation, pest control, and removal of dead or dangerous trees – all of which incur significant costs in manpower and resources. At the same time, budget constraints in city governments mean that resources for tree care are often tight or even shrinking, heightening the need to optimize how and where those funds are used. In practice, this has created pressure to find more efficient ways to monitor tree health^10,23^ and to prioritize maintenance – maximizing outcomes with limited resources without compromising urban forest vitality.

Remote sensing monitoring offers a promising solution to this challenge. Recent advances, especially in the quality of satellite-based approaches, now make it possible to evaluate the health and vitality of urban greenery remotely^24,25^, across entire cities, with unprecedented detail not only in public areas but also in private gardens. Vegetation indices derived from satellite imagery can serve as proxies for plant health^22,26–28^, reflecting factors like leaf greenness and density^29^. High-resolution Earth observation (EO) data provide accurate, spatially explicit indicators of vegetation condition that can be updated frequently and cover areas that would be difficult to assess tree-by-tree from the ground^22^. This approach has proven very effective in natural forests and agriculture, where satellite indices correlate with plant stress and vigor^30–32^, but it has also shown promise in urban environments^24,33^. Applying these indices in cities, however, is not straightforward. The urban setting introduces additional complexity, such as heterogeneous tree species, backgrounds interference from buildings, and fine-scale variability which can hinder direct interpretation of satellite data for individual tree health^22,34^. In essence, while there is an abundance of data from space, the link between these data and on-the-ground arborist assessments is often missing. Translating raw remote-sensing metrics into practical indicators of tree health is currently lacking in many urban forestry applications.

To bridge the gap between scientific data and user-friendly information, a new tool called UpGreen has been developed under a European Space Agency (ESA) Business Applications Programme. It leverages satellite Earth observation data to evaluate the vitality and stress of urban vegetation and estimate the ecosystem services that greenery provides. By using satellite imagery, the tool can assess vegetation health consistently at a global scale, making it a widely applicable solution. Importantly, the use of satellite data makes the approach cost-effective and scalable: assessments can be updated city-wide on a regular schedule, reaching also private gardens, something that traditional ground surveys could never achieve with the same frequency or budget.

In this study, we present an interpretation of the UpGreen tool with real-world arboricultural ground data in two contrasting city environments: Lisbon and Copenhagen. These two cities were chosen for their markedly different climates and urban forestry contexts—Lisbon representing a warm Mediterranean city with hot, dry summers, and Copenhagen representing a cooler Northern European city with a temperate climate and sufficient rainfall—in order to robustly test the tool’s performance under diverse conditions. Professional arborists conducted field assessments of tree health in both cities, providing “ground truth” data on tree vitality, which we then compared with the satellite-derived indices represented by UpGreen outputs. This comparison not only tests the accuracy of the tool’s evaluations of vegetation health but also helps refine the interpretation keys that link specific vegetation index values to on-the-ground tree conditions.

The aims of this study are twofold: first, to evaluate the accuracy of vitality and stress estimation output of the UpGreen tool; and second, to provide practical interpretation guidelines for vegetation indices, translating satellite data into arboricultural terms.

## Materials and methods

### UpGreen methodology overview

UpGreen is a novel satellite-data-driven product developed under the ESA funding, assessing the state and performance of greenery in cities, aiming to help municipalities focus costs on greenery maintenance effectively. It combines satellite data and advanced modelling of greenery behavior in a complex assessment. Computation consists of several steps:

#### Segmentation

Individual tree crowns are detected and delineated via U-Net Neural Networks^35^ using 30 cm aerial imagery in Lisbon. Optimal parameter tuning was done via a randomized search-based algorithm. Polygons of tree segments were then filtered by compactness and area limit. Tree segments in Copenhagen were obtained from municipal sources.

#### Land Surface Temperature (LST)

Computation is based on thermal bands of Landsat 7, 8, and 9 imagery, keeping only clear observations. Computation of temporal statistics for 3-year-long periods is performed.

#### Chlorophyll concentration

Time series of spectral indices, Enhanced Vegetation Index (EVI^22^, and Normalized Difference Red Edge index (NDRE^36^ were computed per observation (tree segment) from PlanetScope 4- and 8-bands data. All available values were extracted, producing time series from 2020 to 2024 per segment. Each observation was aggregated as a zonal median for larger segments and as a value under the sample point for smaller segments. Depressions were filtered out, and the remaining temporal data points were interpolated and smoothed with the Savitzky-Golay filter. Growing season integrals per year were computed.

#### Stress

Stress is conducted as a basic multi-criteria overlay analysis of stress factors, weighted equally. Each compound gets a relative value (0–1) according to the level of stress; maximum stress is thus 3. The combination index comprises three stress factors.

1) Heat stress derived from LST, identifying areas where temperature exceeds 40 °C for a total of seven days during the peak growing season (May to July). If the heat stress is present, the segment is assigned 1 stress point;

2) Proximity to roads is used as a proxy for stress caused by air and soil pollution, soil compaction, soil salinization, and water availability. It can also partially cover light pollution stress from streetlights. It represents how close a given tree segment is to roads, while also taking into account the size of the roads and level of traffic through Open Street Maps (OSM) “highway” classification system. In OSM, the highway tag categorizes roads according to their functional role within the transport network, reflecting their physical characteristics, relative importance, and typical traffic load. The presence of each type of road is checked in three buffer zones (10 m, 50 m, 100 m) and assigned stress points according to the Table 1. Each road is accounted for the smallest buffer zone only. The maximum value of stress is 5 (if segment gets more points, then is assigned value 5), for the purpose of total stress index computation, the value is divided by 5;

**Table 1:** Table of stress points assigned to each tree according to the presence of the Open Street Maps “highway” road type in three buffer zones.

	10 m	50 m	100 m
motorway	4	3	2
trunk	4	3	2
primary	3	2	1
secondary	2	2	1
tertiary	2	1	1
unclassified	1	1	1
residential	1	1	0
motorway link	4	3	2
trunk link	4	3	2
primary link	3	2	1
secondary link	2	2	1
tertiary link	2	1	1
living street	1	0	0
service	0.5	0	0
pedestrian	0.2	0	0
track	0.1	0	0
bus guideway	1	1	0
escape	0	0	0
raceway	0	0	0
busway	1	1	0

3) Water Deficit Stress: Based on the predicted values from climate models (Euro-CORDEX ERA5), water deficit stress was defined as occurring when at least one of the following conditions is met. If the drought stress is present, the segment is assigned 1 stress point:

- The maximum Vapor Pressure Deficit (VPD) exceeds 8 hPa^37^ for at least 21 days during the peak season. VPD is considered a proxy for drought stress as it forces a tree to transpire and, at the same time, rises with decreasing water availability in the soil.
- There are at least 14 consecutive days without rainfall (less than 0.5 mm/day).
- The total precipitation over the last 30 days is less than 10 mm.

UpGreen data.

To validate the performance of the UpGreen tool, we use computed photosynthetic activity derived from EVI and NDRE indices and stress parameter (Stress_UG_) computed as an average stress level of the last three seasons (2022–2024). For the purposes of analyses, it is divided into categories by 0.25 assigned levels 0–10 (10 is the highest stress). There were no observations in the lowest category of stress in any of the two cities. The number of observations in each category is listed in Table 2. The low number of observations in some categories, particularly in conifers, made statistical testing impossible; therefore, interpretation is provided only for those cases where the trend is obvious.

**Table 2:** Number of observations in each category of stress for broadleaves and conifers in Copenhagen and Lisbon.

Stress level	1	2	3	4	5	6	7	8	9	10
Copenhagen broadleaves	65	81	47	45	147	1	1	0	1	0
Copenhagen conifers	6	19	6	8	23	0	0	0	0	0
Lisbon broadleaves	0	43	16	19	26	154	6	10	17	91
Lisbon conifers	0	10	9	1	3	30	1	1	4	14

### Validation methodology

For in-situ validation and assessment of trees, we used standardized methods described in the European Tree Assessment Standard and the Czech Tree Assessment Standard SPPK A01 001:2018, which defines vitality as a set of symptomatic manifestations of individuals’ ability to recover and continue living. The standard presents common fundamental practices used in European countries.

Surrounding conditions can cause stress that negatively affects trees’ health and vitality. We assessed air pollution, light pollution, noise levels, soil compaction and composition, water availability, and permeability of surfaces^38^.

Evaluated parameters of in-situ validation:

- Vitality: physiological condition of the tree which is based on visual analysis of crown structure, leaf density and quality, signs of dieback and regeneration, and defines a tree’s capacity to go on living, but also to develop, grow, and regenerate;
- Health condition: mechanical damage, infestation by wood-decaying fungi and/or xylophagous insects, presence of dry thick branches, presence of cavities and exit holes, presence of defective and damaged branching points;
- Perspective: estimated life expectancy of the tree in its habitat, given tree status and habitat limitations;
- Limited space for root growth: proximity to buildings and big collectors or barriers;
- Soil compaction: proximity to buildings and artificial surfaces;
- Water availability: proximity to impermeable surfaces and water runoff;
- Soil salinity: proximity to road in a linear gradient up to 50 m in areas where icing occurs;
- Heavy metals in soil: proximity to road in linear gradient up to 100 m, or area within old city districts;
- Sunlight exposure: the amount of sunlight and sunshine that the tree is exposed to;
- Artificial light pollution: proximity to public lighting or other sources of artificial light;
- Air pollution: proximity to road, considering the blocking by obstacles.

Categories of locations that were selected for data collection to compare levels of stress from different sources are Parks, Roads, Streets, waterfront (Seaside, Riverside), Courtyards, and Brownfields. In every possible combination of categories mentioned above, the goal was to examine at least 10 samples, where possible to find. Only trees that were previously detected and evaluated by aerial scanning were sampled.

### Ground data in copenhagen

**Fig. 1:**
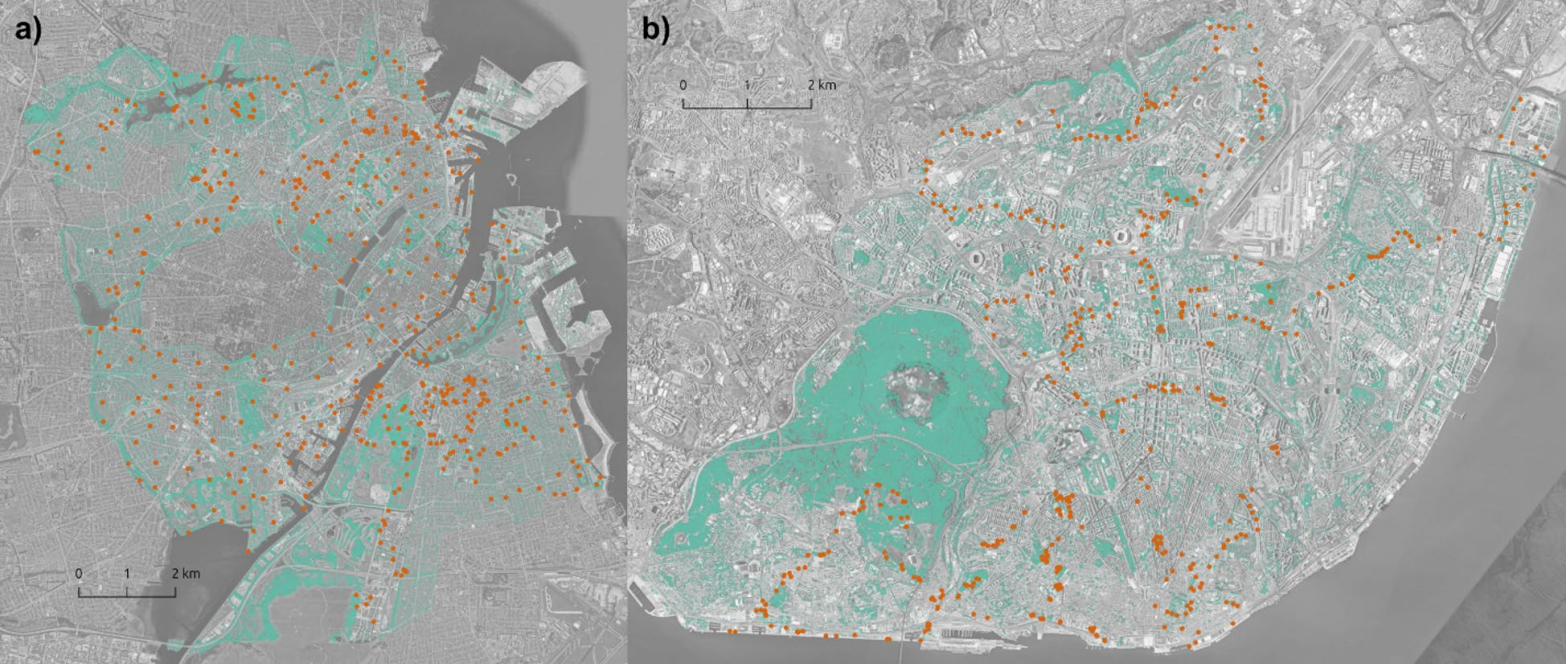
Spatial distribution of sampled trees (orange dots) in (a) Copenhagen and (b) Lisbon. All trees evaluated by UpGreen in both cities are shown in green. Map was generated using QGIS 3.34 Prizren (https://qgis.org/), Google Satellite imagery used in the figure was retrieved through QGIS on 30th July 2025.

Sampling took place in May 2025. Spatial distribution of sampled trees is shown on the map in Fig. 1a. In total, the Copenhagen data set contains 467 data points (evaluated trees) of which:

- 256 trees are of very good or slightly reduced vitality (1),
- 141 trees are of clearly reduced vitality (2),
- 46 trees are of significantly reduced vitality (3),
- 14 trees are of residual vitality (4),
- 10 are dead trees (5).

There is a very small proportion of coniferous trees in Copenhagen. Due to that, it was impossible to collect a representative data set for both broadleaves and conifers. Only three samples are of vitality 3, and one sample of a dead tree; no tree of vitality 4 was found.

### Ground data in lisbon

In situ validation took place in April 2025. Spatial distribution of sampled trees is shown on the map in Fig. 1b. In total, the Lisbon data set contains 478 data points (evaluated trees) of which:

- 200 trees are of very good or slightly reduced vitality (1),
- 177 trees are of clearly reduced vitality (2),
- 73 trees are of significantly reduced vitality (3),
- 27 trees are of residual vitality (4),
- 1 is a dead tree (5).

### Statistical analyses

All analyses were performed in R^39^. Exploratory analysis was done using basic statistical methods such as correlation analysis, linear regression, or ANOVA (one- and two-way) with Tukey’s Honest Significant Difference post-hoc test. Cohen’s d was computed to estimate effect sizes. All significances were evaluated at the level of probability α = 0.05.

Broadleaved and coniferous trees were analyzed separately, as gymnosperm and angiosperm species have different wood structure and physiology^40–42^. In addition, the characteristics such as the amount of chlorophyll and stomata may vary substantially between these groups^43,44^, which can influence their spectral and growth responses. Therefore, the two groups were not directly compared.

Limitations.

Both types of measurements, ground and satellite-derived, are subject to error. Regarding tree height, ground observation is done only through visual inspection, and height is estimated based on neighboring objects of a known height, such as streetlights or building floors. As such, it can bear an error, especially in taller trees of more than 15 m. Satellite-derived data of tree height are estimated from Digital Surface Model and Digital Terrain Model (where available) and smoothed, and thus underestimate real height.

In the case of crown area estimation, a similar situation occurs. As ground estimates assume a circular shape of a crown, in trees with very asymmetric crowns, the estimate can be overestimated. In the case of EO-derived crown area it can bear an error coming from several sources: (1) one large crown can be separated into smaller patches; (2) a few smaller crowns can be identified as a single crown; (3) shades of tree crowns can artificially enlarge the crown area; (4) segmentation comes from older data (2019 in both cities).

Comparison of such datasets needs to be interpreted cautiously; none of them can be considered a definitive reference, and, a lower r^2^ is expected due to a bias in both sets of data.

## Results

### Segment size

The relationship between ground-measured height and satellite-derived height is linear with r^2^ = 0.47 in Copenhagen (in Lisbon, not evaluated due to the missing Digital Terrain Model). In taller trees, ground estimates tend to underestimate height.

Despite all these sources of uncertainty on both sides, the relationship is significant (r^2^ = 0.39 in Copenhagen and r^2^ = 0.26 in Lisbon) and linear, with ground estimates being generally larger at smaller trees than EO estimates.

As DTM/DSM-derived height is reported to underestimate especially lower trees^45^ and ground estimates seem to underestimate tall trees, we use the average of both approaches in further analysis. The size of a tree was approximated as a combination of crown area and height as follows:

$$Size [m]=(crown\_{area} [m^{2}] * height [m])^{1/3}$$

This approximation was compared to Diameter at Breast Height (DBH), and for both conifers and broadleaves in both cities, there was a significant correlation between the two parameters.

Stress.

Stress_UG_ became significantly associated with all eight estimated stress categories observed during ground data collection in Copenhagen, whereas in Lisbon, it was significantly associated with all categories except for soil salinity and soil compaction. Dependencies in Copenhagen were stronger than in Lisbon, as indicated by larger Cohen’s d values (Table 3).

**Table 3:** Cohen’s d effect sizes of lowest vs. highest stress levels in all ground stress categories. Values only for significant dependencies are listed.

	Copenhagen	Lisbon
Space for roots	0.71	0.32
Soil salinity	0.69	
Soil compaction	0.94	
Water availability	0.91	0.33
Metals in soil	1.13	0.96
Sunlight exposure	0.72	0.16
Light pollution	1.12	1.13
Air pollution	1.6	1.10

### Vegetation indices

EVI was compared to NDRE and performed very similarly (r^2^ = 0.72). Both EVI and NDRE were tested for dependence on tree size. For broadleaves, the best-fitting curve for NDRE was a third-degree polynomial (r^2^ = 0.36), which tended to saturate for the biggest trees. The best-fitting curve for EVI was a line (r^2^ = 0.31). EVI, unlike NDRE, does not tend to saturate. Due to that, only EVI was used to further analyze dependencies on arboristic parameters.

EVI shows significant dependence on tree size in both cities. Such an effect is not the focus of this study. Therefore, for linking arboristic parameters to vegetation indices-derived tree performance, we removed the effect of size from the data and further analyzed only residuals of fitted regression lines separately for conifers and broadleaves. Such residuals are interpreted as the performance of a tree compared to an average tree of the same size in the area and further referenced as EVI_res_.

### Copenhagen: Broadleaves

**Fig. 2:**
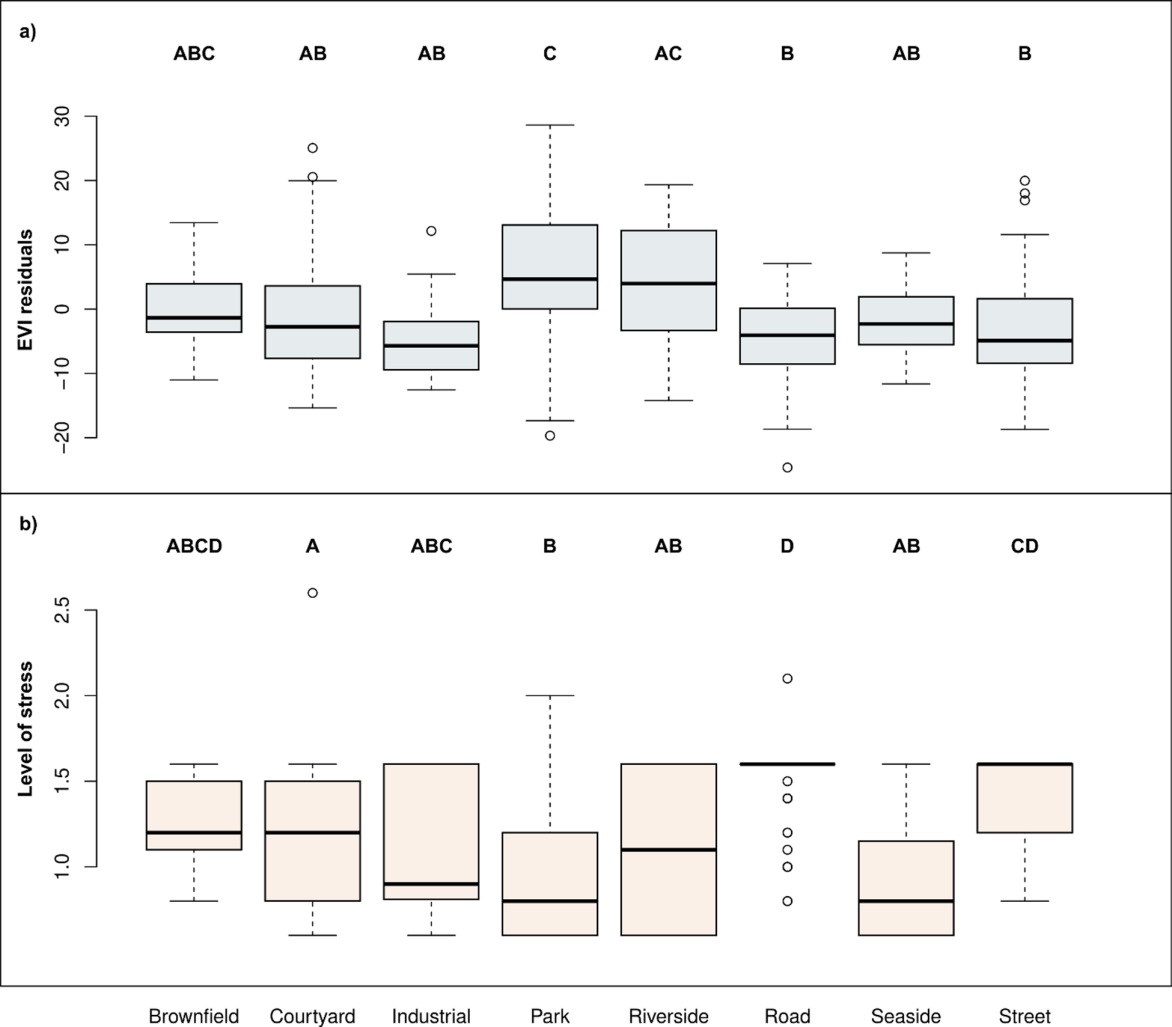
Boxplots showing broadleaved trees in Copenhagen: (a) differences in photosynthetic activity (EVI_res_) depending on categories of location; (b) differences in stress levels depending on categories of location. Statistically significant differences are denoted with letters.

EVI_res_ showed significant differences among location categories (Fig. 2a). The most pronounced contrasts were observed between park sites and more urbanized environments such as roads, streets, and industrial areas, indicating a clear spatial gradient in vegetation condition.

**Fig. 3:**
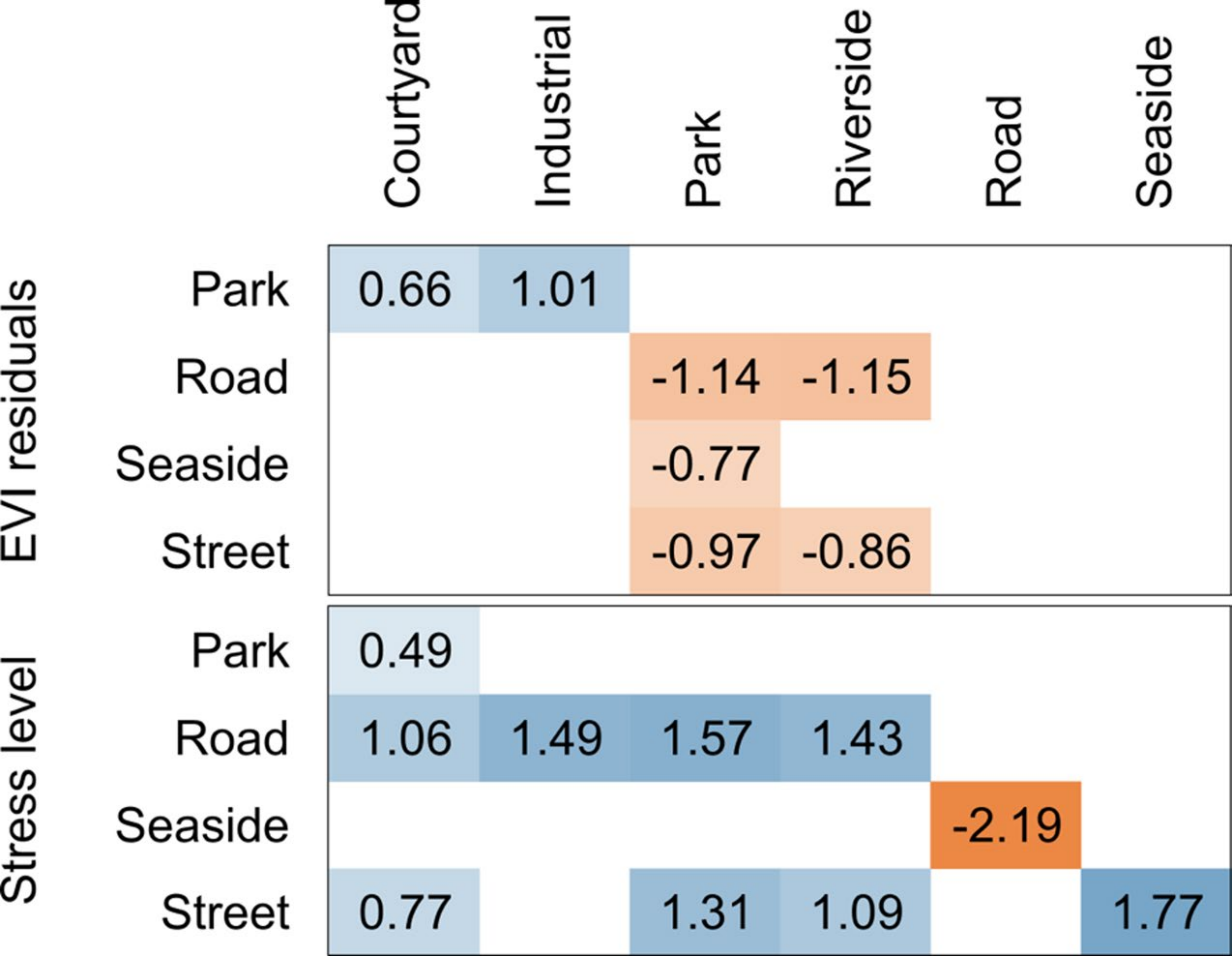
Cohen’s d of statistically significant differences of EVI_res_ and Stress_UG_ between locations.

Consistent with this pattern, Stress_UG_ also differed significantly among location categories (Fig. 2b). Higher stress levels were generally recorded in highly exposed environments (e.g., roads, industrial and seaside sites), whereas parks and courtyards showed lower stress levels. The magnitude of these differences was substantial, as indicated by Cohen’s d (Fig. 3).

**Fig. 4:**
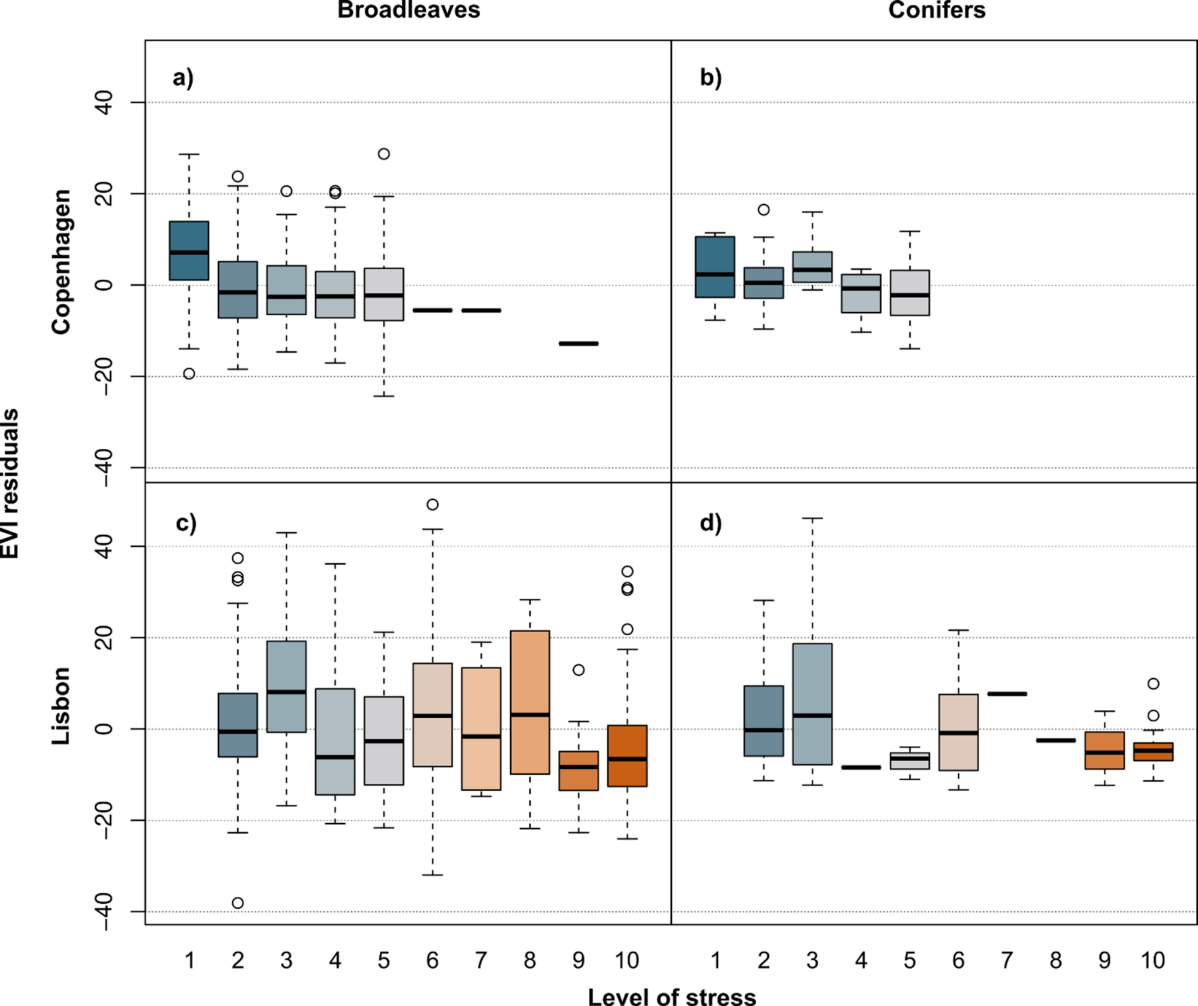
Boxplots showing the dependence of EVI residuals on the level of stress in (a) Copenhagen broadleaves, (b) Copenhagen conifers, (c) Lisbon broadleaves, (d) Lisbon conifers. Categories of stress indicate a stress index divided into categories by 0.25 assigned levels 0–10, with 10 representing the highest stress.

EVI_res_ is significantly dependent on stress: space, compaction, water availability, salinity, metals, sunlight, light pollution, and air pollution. Significant dependence was also found on Stress_UG_, as shown in Fig. 4a. Trees under the lowest level of stress have significantly higher activity than trees under any level of stress (Cohen’s d 0.96).

**Fig. 5:**
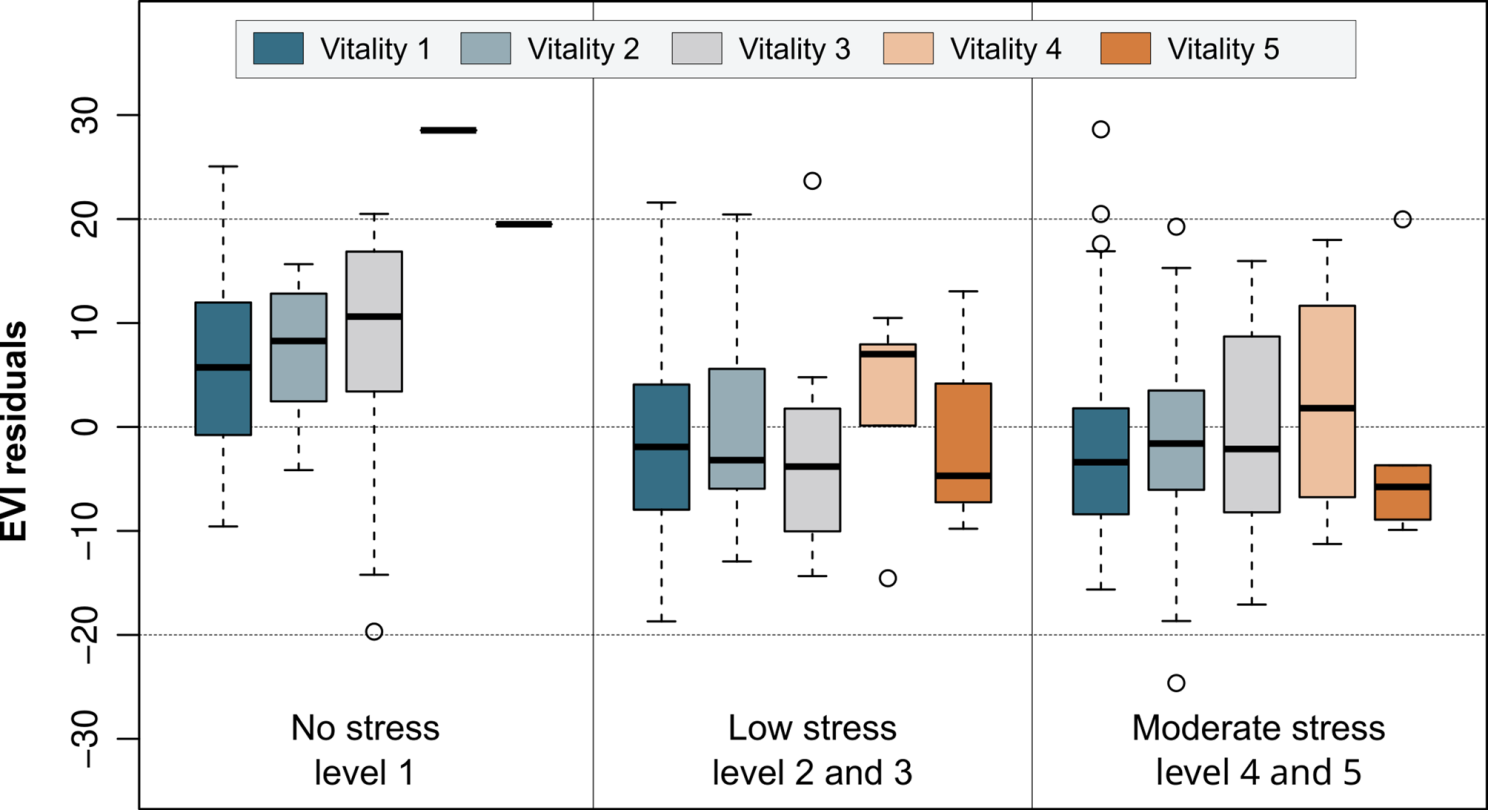
Boxplots of EVI residuals in dependence on vitality and stress level for broadleaves in Copenhagen. Categories of stress indicate a stress index divided into categories by 0.25 assigned levels 0–10, with 10 representing the highest stress.

Chlorophyll concentration tends to increase with decreased vitality in all levels of stress (Fig. 5). In more stressed trees, the increase occurs only at the latest stages of vitality loss, while in less stressed trees, the increase in activity is apparent already in slightly reduced vitality.

Two-way ANOVA of dependency of residuals on stress and vitality became significant for stress and close to significant for vitality (*p* = 0.07).

### Copenhagen: Conifers

Due to the small number of coniferous trees in Copenhagen, the sample size is limited, and thus, the possibilities of testing are also limited. Only in the location categories of Courtyard, Park, and Street are there more than 5 observations in each category. Among these categories, there are no significant differences in EVI_res_.

EVI_res_ dependence on Stress_UG_ is shown in Fig. 4b; differences are not significant. Stress_UG_ was also tested among the same location categories. The only significant difference was found between Park and Street (Cohen’s d −0.94); the rest were close to significant.

EVI_res_ follow the same pattern in dependence on age as observed in broadleaves. However, testing is not possible due to only two observations in age category 2 and only one observation in age category 5. Age category 1 is missing.

The same applies to dependence on arboristic stress categories. There are obvious differences, but it is not possible to test due to the small number of observations in most categories.

Testing dependencies on vitality is not possible due to the missing data in categories of lower vitality.

Lisbon: Broadleaves.

Despite residuals being visually dependent on location, differences become statistically insignificant. This can be explained by the level of stress.

Trees in Lisbon are much more stressed in general than in Copenhagen. Stress level below 0.75, which became the threshold level for significant difference in Copenhagen, is not found in any tree in Lisbon due to very harsh conditions during peak growing season, especially due to drought and heat stress (Fig. 4c, d). The two highest stress categories (9 and 10) have significantly lower photosynthetic activity than the other stress categories (Cohen’s d 0.54).

To support the theory of trees being extremely stressed by heat and drought, the dependency on light/shade was tested. Category 3 (shaded trees) has significantly higher activity than the other two categories (Cohen’s d 0,49). Differences in Stress_UG_ among locations are not significant, supporting the explanation of overall high stress levels.

**Fig. 6:**
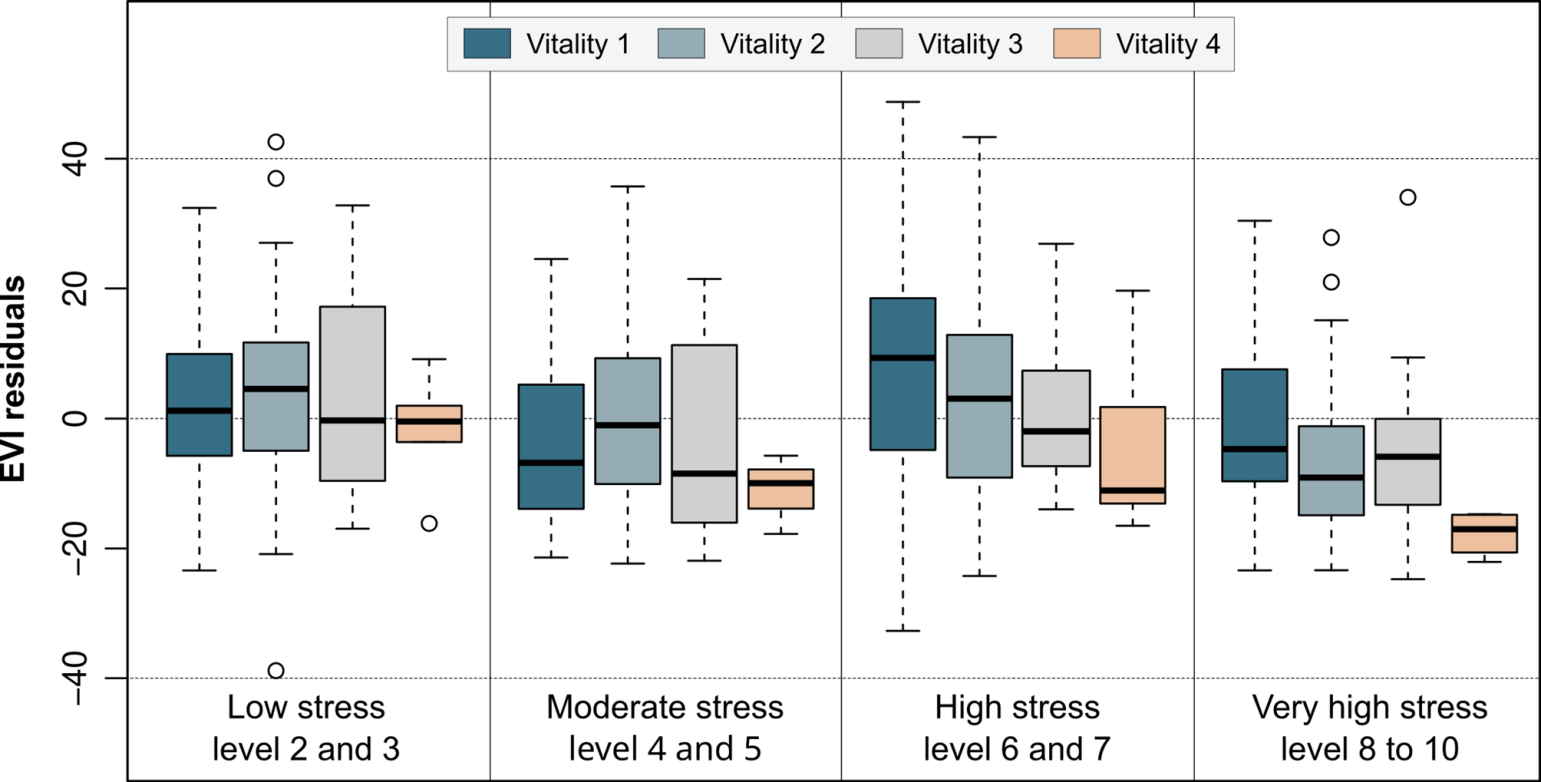
Boxplots of dependence of EVI residuals on vitality in different stress categories for broadleaves in Lisbon. Categories of stress indicate a stress index divided into categories by 0.25 assigned levels 0–10, with 10 representing the highest stress.

Unlike in Copenhagen, in Lisbon broadleaves EVI_res_ are significantly dependent on vitality regardless of the stress level, while the difference is more pronounced in higher stress (Fig. 6).

Two-way ANOVA of the dependence of residuals on stress and vitality became significant for both variables.

Unlike in Copenhagen, in Lisbon, residuals were not dependent on age.

### Lisbon: Conifers

There are only a few samples in most location categories. Thus, only differences among Courtyard, Park, and Street were tested. The only significant difference was found between Park and Street (Cohen’s d 0.33). This is also in agreement with Copenhagen’s broadleaves.

Dependency on age is impossible to test due to missing data in categories 1 and 5, and only one sample in category 2. As in Copenhagen’s broadleaves, there is no difference between ages 3 and 4.

There is a decreasing trend in reaction to stress. While in broadleaves the main growing season occurs in spring/summer, in adapted Mediterranean conifers the peak is usually in winter or spring. Compound stress factor Stress_UG_ considers water availability throughout the season, while these conifers benefit from relatively humid conditions in winter, while not harmed by drought in summer. Therefore, the most significant stressors are those connected with soil compaction, pollution, or general water availability (e.g., due to paved soil). EVI_res_ gradually decreases with stress levels of space for root growth, soil compaction, water availability, and air pollution, as collected during ground sampling. The only significant difference was found between stress levels 1 and 3 in space for root growth; all other differences were close to significant (*p* = 0.07 in all cases).

Stress_UG_ levels differ among location categories as expected but were not tested due to the small number of observations.

As in Copenhagen, testing dependencies on vitality is not possible due to the missing data in categories of lower vitality. However, a visual check shows a similar pattern as Copenhagen’s broadleaves: the lower vitality, the higher activity.

## Discussion

### Data set

The data set includes data from two cities with very contrasting conditions, defining close-to-edge conditions within Europe. The contrasting conditions support the generality of the used methodology in UpGreen; however, interpretations cannot be simply extrapolated to mid-latitude cities because central conditions are not covered satisfactorily. Thus, further testing on smaller and/or Central European cities is needed. Results show general patterns in trees’ reactions to unnatural conditions. The magnitude of reactions in each studied aspect differs according to the adaptability and adaptation state of the city ecosystem to extreme and changing conditions.

Not only climate conditions, but also the tree demography differs substantially between the two cities. In Lisbon, tree population renewal occurs more gradually and continuously, with individual losses being steadily offset by new plantings. In contrast, Copenhagen experienced a substantial Ash die-back in the 00’s^46^, which was widely used in city planting before (personal communication, Zwergius Teilmann S., 2024). The dieback was followed by more concentrated replanting efforts; thus, many trees are young or juvenile. Consequently, responses to stress can differ greatly in general. Young trees are generally more flexible but also more vulnerable e, while older trees have large non-structural carbohydrate pools and better developed root systems to survive extended stress but are less adaptable^47–51^. In Copenhagen, we can see a more uniform reaction to stress, while in Lisbon, the response is more variable.

The dependency of EVI_res_ on tree size might be partially caused by the coarse resolution of input data and mixing signals in smaller trees (and thus artificial). A further possible explanation is that bigger (older) trees have generally higher Leaf Area Index^52^.

### Link to vitality and stress

Vitality definitions differ across fields and countries^53^. While in forestry and ecology, the definition is usually broad and interconnected with stress^54^, in arboriculture and horticulture, the vitality is usually understood as a set of symptomatic manifestations of individuals’ ability to recover and continue living^55,56^. Such symptoms, however, can manifest significantly later than the tree starts losing vitality in a broader sense^57,58^. Through satellite scanning of greenness, we can see very small year-to-year changes that may or may not be assigned solely to weather conditions^59^. Despite all inconsistencies in definitions across fields, our study shows a clear potential of satellite data-derived vegetation indices to serve as proxies of vitality and stress of urban trees, as defined in arboriculture on an individual-tree level.

In Copenhagen, we have shown a strong dependence of EVI_res_ on location and Stress_UG_. Those represent an independent stress level evaluation. While in Copenhagen stress levels are generally very low, in Lisbon they are mostly extreme. In the context of different species composition in both cities, insignificant differences in EVI_res_ in response to stress level, the possible explanation could be a shifted peak of the growing season to earlier months as an adaptation to such harsh conditions, while the stress index reflects mainly the summer months. Another possible explanation for the lower levels of significance in Lisbon could be higher uncertainty in the data caused by a longer vegetation season and the presence of understorey. In Copenhagen, with decreasing vitality, trees react with their greenness differently than in Lisbon. Trees with lowered vitality show higher greenness. The effect is more pronounced in trees under no stress. In stressed trees, the effect is also apparent but much weaker. In Lisbon, the stress conditions are harsher. In stress levels 6–10, trees of good vitality show much higher greenness than in lower stress levels. A possible explanation lies in the trees’ demographic differences. Predominantly young trees in Copenhagen may be more resistant and adaptable to stress conditions, not reacting to it with decreasing vitality in a short time^60^. Another explanation would be in osmotic adaptation and the non-structural carbohydrates (NSCs) accumulation and remobilization^48^. Non-structural carbohydrates act as osmoprotectants and drivers of water flux, helping to maintain a sufficient turgor gradient for water transport^50^. However, systematic research on urban trees’ stress reactions is largely missing, and there is support in the literature for mechanistic interpretation only for natural ecosystems (forests). As urban trees are predominantly exposed to multiple sources of stress at higher levels than trees in natural conditions^15^, reactions can thus be different. There are only a few studies from Europe on the topic of drought or heat stress, most of them being case studies located in Germany and Sweden, none of which looks at the effects of stress on tree vitality or health. The only study comparing physiological heat stress reactions of trees in urban and natural forest stands^61^ shows that chlorophyll content rises with increased heat in urban areas, unlike in forests for *Quercus cerris*, supporting more general results in our study.

### Sources of uncertainty

In Lisbon, the growing season is shifted to earlier months. The limiting factor is not temperature, like in Copenhagen, but water availability^62–64^. In late winter/early spring (around February), the understorey can already be very tall and green, while in summer and autumn it stays brown. That can result in a less accurately derived baseline for EVI integral computation and thus increase the variability of the results. In Copenhagen, the peak growing season is very stable across species due to temperature limitations^65,66^. Thanks to that fact, we could evaluate and compare only months with the main growth activity of the trees, while filtering out the effect of understorey greening before this phase. In Lisbon, the situation is much more complicated due to the inverted seasonal pattern of physiological activity (with the peak activity in winter) of some coniferous species^67–70^. Thus, the effect of the understorey could not be filtered out like in Copenhagen.

Further limitations relate to termporal and biological uncertainty. Vitality assessed by arborists during on-site data collection might not reflect the state of the tree that was measured by satellite-derived indices in previous years^59,71^. Vitality may change very rapidly; however, it can stay stable (even in a degraded condition) for many years. Interpretation of results is additionally constrained due to the absence of knowledge of tree species-level information, since reactions to stress may substantially differ among species due to traits such as an/isohydricity^72^, resistance to air pollution, xylem vulnerability, or rooting depth^73–76^. Missing DTM in Lisbon does not limit the results in our case, as we analysed only trees that were assessed during a field survey. However, using satellite-derived vegetation indices for the overall assessment of tree health and vitality within any city without any ground data might require the knowledge of trees’ height to effectively filter out low shrubs.

Finally, as EVI (and vegetation indices in general) show chlorophyll presence in leaves, not its photosynthetic activity, it would be effective to distinguish between real photosynthesis and the passive presence of chlorophyll in leaves by fluorescence. Remote sensing options are, however, very limited. Currently available data have a spatial resolution in kilometers, which is unapplicable in an urban environment. According to our findings, estimated stress conditions could be a good proxy to distinguish between the presence and activity of chlorophyll.

## Conclusions

Despite all the uncertainties in the data on both sides (satellite and ground), the results show general patterns and significant dependencies, which should not be overlooked. We identified patterns in trees’ response to stress and observed dependencies of greenness on vitality in agreement with current knowledge. Despite further research on urban trees being needed, our results can be translated into practice and improve the explainability of satellite data-derived vegetation indices in arboricultural terms. Moreover, we have proved an easy and generally applicable stress index to be consistent with ground observations. As such, this study provides a missing guide to municipalities and greenery maintenance groups in cities on how to interpret satellite data-derived vegetation indices. We found significant relationships between vitality and stress; however, their interpretation depends on local environmental conditions.

## Data Availability

The geopackage data that support the findings of this study are available in Zenodo repository with the identifier https://doi.org/10.5281/zenodo.17510513.
